# Catarrh

**Published:** 1892-02

**Authors:** 


### CATARRH.

People who are subject to catarrhal ailments have special need to be particular in regard to their feet covering ; they should see to it that their feet are comfortably clad, their shoes should have substantial soles, and should come well up the ankles, and not be laced or buttoned tight. Light merino stockings or half-hose may be sufficient for warmth, but whenever by reason of much exercise the feet have become damp, and especially if the leather has absorbed wet, it is wise for a change to be made in both stockings and shoes.

